# *Dendropanax morbifera* Leaf Extracts Improved Alcohol Liver Injury in Association with Changes in the Gut Microbiota of Rats

**DOI:** 10.3390/antiox9100911

**Published:** 2020-09-24

**Authors:** Taekil Eom, Gwangpyo Ko, Kyeoung Cheol Kim, Ju-Sung Kim, Tatsuya Unno

**Affiliations:** 1 Subtropical/Tropical Organism Gene Bank, SARI, Jeju National University, Jeju 63243, Korea; taekil7@hanmail.net; 2 Faculty of Biotechnology, College of Agriculture & Life Sciences, SARI, Jeju National University, Jeju 63243, Korea; rhkdvy1004@gmail.com; 3 Majors in Plant Resource and Environment, College of Agriculture & Life Sciences, SARI, Jeju National University, Jeju 63243, Korea; cheolst@jejunu.ac.kr (K.C.K.); aha2011@jejunu.ac.kr (J.-S.K.)

**Keywords:** *Dendropanax morbifera*, hepatoprotective, leaf extract, liver damage, alcoholism, oxidative damage

## Abstract

This study evaluated the protective effects of *Dendropanax morbifera* leaf (DML) extracts in the liver due to excessive ethanol consumption. Our results showed that the ethanol extract had better antioxidant activity than the water extract, likely due to the higher levels of total flavonoid and phenolic compounds in the former. We found that the main phenolic acid was chlorogenic acid and the major flavonoid was rutin. Results from the animal model experiment showed concentration-dependent liver protection with the distilled water extract showing better liver protection than the ethanol extract. Gut microbiota dysbiosis induced by alcohol consumption was significantly shifted by DML extracts through increasing mainly *Bacteroides* and *Allobaculum*. Moreover, predicted metabolic activities of biosynthesis of beneficial monounsaturated fatty acids such as oleate and palmitoleate were enhanced. Our results suggest that these hepatoprotective effects are likely due to the increased activities of antioxidant enzymes and partially promoted by intestinal microbiota shifts.

## 1. Introduction

The worldwide increase in alcohol consumption has led to alcoholic liver disease (ALD) accounting for more than 5% of all diseases, with more than three million deaths being estimated to be from ALD. ALDs include alcoholic fatty liver disease, alcoholic hepatitis, and alcoholic cirrhosis, and these can later develop into liver cancer [1,2].

The liver is an organ that absorbs, metabolizes, and stores various substances that enter the body, with 80% of the alcohol consumed by humans being detoxified by the liver. When alcohol is consumed, it is absorbed from the small intestine and metabolized by enzymes such as alcohol dehydrogenase (ADH) and aldehyde dehydrogenase (ALDH) in the liver, but when excessive alcohol consumption exceeds the metabolic ability of ADH and ALDH, the expression of Cytochrome P450 2E1 (CYP2E1) enzymes is induced, leading to alcoholic metabolism [3]. This can lead to production of acetaldehyde or reactive oxygen species (ROS) and toxic substances, causing oxidative damage and inflammation to liver cells, resulting in ALD [4]. ROS produced by excessive alcohol consumption are known to be removed by antioxidant enzymes to prevent oxidative damage caused by alcohol. Antioxidant enzymes such as catalase (CAT), superoxide dismutase (SOD), glutathione *S*-transferas (GST), and glutathione reductase (GR) play a direct role in removing ROS [5].

The intestines are normally home to various types of bacteria, with the term “gut microbiota” referring to the various microorganisms that exist in the human gut. Although the functions of the gut microbiota are not completely known, their effects on human health have recently been actively studied [6,7]. In particular, the gut microbiome is involved in inhibiting the growth of pathogenic bacteria, stimulating the production of total and pathogen-specific mucosal IgA, nutrient production in mucosal cells, the development and regulation of the immune system, and immunological resistance. Thus, the gut microbiome appears to play an essential role in maintaining human homeostasis by engaging in bidirectional interactions with the host [8]. Gut microbiomes vary depending on race, age, dietary habits, and drug use, and the impact of these factors on the health of the host due to changes in the gut microbiome is referred to as dysbiosis [9]. Several recent studies have shown that liver damage caused by alcohol consumption and gut microbiota interact very closely with each other, which is referred to as the gut-liver axis [10,11,12].

Recently, many studies using various medicinal plant resources have been conducted on foods and medicines that prevent liver damage due to excessive alcohol intake [13,14]. However, studies on changes in the gut microbiome caused by medicinal plants and their effects on the prevention of liver damage are insufficient. *Dendropanax morbifera*, which belongs to the Araliaceae family, is a medicinal plant that grows on the southern coast of Korea and is known to have various physiological functions such as anti-inflammatory, anti-cancer, anti-diabetic, and immune regulatory effects [15,16,17,18]. In addition, a previous study conducted by our research team showed that *D. morbifera* extract, with its maximal antioxidant activity, inhibited liver damage caused by oxidative stress [19]. Therefore, this study aims to verify the prevention of alcoholic liver damage by *D. morbifera* extract and to analyze its relationship with the gut microbiome, thereby examining its mechanism of action.

## 2. Materials and Methods

### 2.1. Extraction

The *D. morbifera* leaves and stems used in the experiment were purchased from Jeju Hwangchil (Jeju, Korea), and were dried in the shade, crushed, and stored in a refrigerator. To extract the *D. morbifera* leaves and stems, 1 L of 70% ethanol or distilled water was added per 100 g of the sample, followed by three rounds of extraction for 3 h at 80 °C. After the extraction, the sample was filtered under reduced pressure, concentrated using a rotary evaporator (Hei-VAP Precision, Heidolph, Schwabach, Germany), and dried using a freeze dryer (SCANVAC, Stockholm, Sweden), then used in the experiment.

### 2.2. Total Polyphenol and Flavonoid Contents

The polyphenol and flavonoid compounds contained in the extracts were quantified using the Folin-Ciocalteu method and the aluminum chloride method as previously described [20].

### 2.3. Antioxidant Activity

The antioxidant activity of the extracts was compared and evaluated using the 1,1-diphenyl-2-picrylhydrazyl (DPPH) radical scavenging assay, as well as the total equivalent antioxidant capacities (TEAC) and ferric reducing antioxidant power (FRAP) methods as previously described [19].

### 2.4. HPLC Analysis of Phenolic Acid and Flavonoid Components of D. morbifera Extracts

Flavonoid and phenolic acid contents were quantified using the prominence HPLC system with an SPD-M20A PDA detector (Agilent infinity 1260 series, Munich, Germany). The analysis was performed with a Triart C-18 column (250 mm × 4.6 mm, 5 μm) from YMC Co., Ltd. (Tokyo, Japan). The flavonoid analysis conditions are column temperature 35 °C, flow rate 0.8 mL/min, injection volume 10 μL and detection wavelength 280 nm. The gradient program was designed as follows: Distilled water containing 0.1% trifluoroacetic acid (Sigma-Aldrich, Steinheim, Germany) was mixed with acetonitrile (Sigma-Aldrich) containing 0.1% trifluoroacetic acid with the concentrations increased from 10% to 20% for the first 5 min, held for 20 min, 20% to 25% for 10 min, held for 15 min, 25% to 30% for 5 min, held for 10 min, 30% to 60% for 5 min, held for 5 min, 60% to 80% for 5 min, and held for 5 min (total 80 min).

The phenolic acid analysis conditions are column temperature 45 °C, flow rate 1.0 mL/min, injection volume 10 µl and detection wavelength 245 nm. The gradient program was designed as follows: Distilled water containing 0.1% formic acid (Sigma-Aldrich) for 4 min, and mixed with methanol (Sigma-Aldrich) containing 0.1% formic acid with the concentrations increasing from 0% to 15% for 10 min, held for 3 min, increased from 15% to 16.5% for 7 min, 16.5% to 18% for 4 min, 19% to 25% for 2 min, 25% to 28% for 6 min, 28% to 30% for 2 min, held for 3 min, increased from 30% to 40% for 5 min, 40% to 48% for 2 min, 48% to 53% for 5 min, 53% to 60% for 10 min, 60% to 70% for 2 min, and held for 5 min (total 70 min).

### 2.5. Animal Experiments

Five-week old Sprague Dawley (SD) rats were purchased from Daehan Biolink (Eumsung, Korea) and used after a one-week acclimation period. The animals were kept in a room with a temperature of 20–22 °C, a humidity of 50%, and a 12 h light-dark cycle. All experiments were approved by the Jeju University Institutional Animal Care and Use Committee (IACUC) (Approval number: 2016-0056).

In order to see whether the *Dendropanax morbifera* leaf (DML) extracts protected against alcoholic acute liver damage, the experiment was conducted in 8 groups as follows. Dose of ethanol and DML extracts were determined as previously described [19,21]. An overview of the experimental design is outlined in Figure S1. Before the feeding trial, all mice were acclimated for 7 days by feeding normal diet ad libitum. Feeding trial was conducted for 10 days. At the first day, animals were divided into 3 groups and fecal materials were collected. (Control (CTL), fed normal diet during the trial; Ethanol, fed normal diet for 10 days and ethanol was supplied from day 8 to day 10; and DML extracts group, fed normal diet with various concentrations of DML extracts for 10 days and ethanol was supplied from day 8 to day 10). Ethanol (5 g/kg) and DML extracts (100, 300, 500 mg/kg) were administered orally. On the 10th day, 8 h after the last ethanol intake, fecal materials were collected, and rats were sacrificed for dissection.

### 2.6. Serum Biochemical Analysis

The blood samples obtained were left on ice for 20 min and then centrifuged at 570× *g* for 10 min, after which the upper layer of the serum was collected. The separated serum was stored at −80 °C prior to use in the experiments. Serum ethanol and acetaldehyde concentrations were measured and quantified using an ethanol assay kit (Megazyme, Bray, Ireland) and an acetaldehyde assay kit (Megazyme, Bray, Ireland), respectively. Serum aspartate aminotransferase (AST) and alanine aminotransferase (ALT) concentrations were measured using an automated hematology analyzer.

### 2.7. Antioxidant Enzyme Activity

For the analysis of antioxidant enzyme activity in liver tissue, a sample of liver tissue was homogenized using a homogenizer in 50 mM phosphate buffer (pH 7.4). The homogenized suspension was centrifuged at 3000× *g* for 20 min at 4 °C, and the supernatant was used to measure the enzyme activity. CAT, SOD, GR, and GST were considered as oxidative stress markers [5] and measured as follows: SOD activity was analyzed using the McCord & Fridovich method [22]; and CAT activity was determined using the protocol by Aebi [23]; GST and GR activity was measured using a protocol by Koneru et al. [24]. The amount of protein was quantified using bicinchoninic acid (BCA) to measure the enzyme activity.

### 2.8. Hepatic Histopathological Observation

For histopathological observations, a portion of the middle lobe of the liver was dissected and fixed in 10% buffered formalin solution (Sigma-Aldrich, Steinheim, Germany) for 24 h. After the fixation, the tissue was embedded in paraffin, cut to a thickness of 5 μm, and stained with hematoxylin (Sigma-Aldrich, Steinheim, Germany) and eosin (Sigma-Aldrich, Steinheim, Germany) (H&E) for observation using an optical microscope with 100-fold magnification.

### 2.9. Analysis of Gut Microbiota

For microbial community analysis, the V4 hypervariable region of the 16S rRNA gene was amplified, and a library for Illumina Miseq (250 bp × 2) was constructed with two-step PCR. Briefly, first PCR was performed using a KAPA HiFi HotStart ReadyMix PCR kit (Roche, South San Francisco, CA, USA) as follows: 95 °C for 3 min, 25 cycles of 95 °C for 30 s, 55 °C for 30 s, and 72 °C for 30 s, and 72 °C for 5 min. The obtained PCR products were further purified using a HiAccuBead (AccuGene, Seoul, Korea). The purified PCR products were again subjected for PCR to attach barcode-sequences. After PCR products were purified in the same manner, an equimolar of the final PCR amplicons were pooled and sent for sequencing with MiSeq according to the manufacturer’s instructions at Macrogen Inc. (Seoul, Korea).

Sequence data was analyzed using MOTHUR [25]. In brief, raw reads paired-end assembly was done with make.contigs, and then aligned to the SILVA Database [26]. After eliminating the singleton, the pre.cluster MOTHUR subroutine was performed to correct the error for rare sequences. Chimeric sequences were detected using VSEARCH [27]. Taxonomic classification was done using Ribosome database project (RDP version 16) database [28]. Sequences classified to undesired taxa (i.e., Chloroplast and Mitochondria) were removed using remove.lineages MOTHUR subroutine. Clustering was performed with 97% similarity using Opti.clust [29] and designated as operational taxonomic units (OTUs). Number of reads per sample was normalized to 20,000 for downstream analyses. Species richness and evenness were evaluated using Chao [30] and Shannon indices [31], respectively. The non-metric multidimensional scaling (NMDS) analysis was conducted based on the Bray-Curtis distance [32]. The linear discriminant analysis effect size (LEfSe) [33] was used to identify significantly increased or decreased taxa after the DML extracts treatments. Analysis of molecular variance (AMOVA) was applied to test significant difference between microbiota. Metabolic activities were predicted using Phylogenetic Investigation of Communities by Reconstruction of Unobserved States (PICRUSt) 2 [34] and abundance comparison was performed using the ALDEx2 package in R software (https://www.r-project.org/) [35]. Spearman correlation analysis was performed to estimate associations between the LEfSe-selected OTUs and ALDEx2-selected predicted metabolic activities.

### 2.10. Statistical Analysis

Analysis of all data is expressed as ± standard deviation, and significance was determined using ANOVA and verified using the Duncan test. Minitab ver. 17 (Minitab Inc., IL, State College, PA, USA) was used for all statistical analysis.

## 3. Results

### 3.1. Total Polyphenol and Flavonoid Contents

The total polyphenol and flavonoid contents were higher in *D. morbifera* leaf (DML) extracts than stem extracts, and higher in the 70% ethanol (EtOH) extracts than the water extracts (Table S1). Flavonoid content specifically was 1.5 times higher in 70% EtOH than in water for leaf extracts, and seven times higher in 70% EtOH than in water for stem extracts.

### 3.2. Antioxidant Activity

Three widely used antioxidation experiments were used to measure the antioxidant activity of DML and stem extracts, and the results are shown in Table S2. The DPPH radical scavenging assay is the simplest method of measuring antioxidant activity, wherein electrons or protons are provided to unstable DPPH radicals, and the ability to scavenge the radicals is measured. DPPH radical scavenging assays showed similar radical scavenging activities for water and EtOH extracts, but leaf extracts showed more than twice the radical scavenging activity of stem extracts. FRAP activity measures antioxidant activity based on the reducing power of the sample, and samples with a large reducing power are considered to have excellent antioxidant activity, as they can effectively reduce ROS with high oxidation states. The results from the FRAP activity assays were consistent with those obtained from the DPPH radical scavenging assay, with the leaf extract demonstrating more than twice the reducing power of the stem extract, and the water and ethanol extracts having similar reducing powers. TEAC activity compares and evaluates the antioxidant activities of samples with an equal amount of Trolox, which is used as an antioxidant. The TEAC value showed a similar trend, but the difference between the leaf and the stem extracts was not as significant as observed with the DPPH and FRAP assays. The antioxidant activity evaluated was in the order of *D. morbifera* leaf ethanol extract (DMLEE), *D. morbifera* leaf distilled water extract (DMLDE), *D. morbifera* stem ethanol extract (DMSEE), and *D. morbifera* stem distilled water extract (DMSDE), which was consistent with the total phenolic and flavonoid contents.

### 3.3. Flavonoid and Phenolic Acid Analyses of D. morbifera Leaf Extract

Content analysis was performed for 18 flavonoids and 12 phenolic acids using HPLC (Figure S2). A smaller number of flavonoids were identified in the water extracts compared to the ethanol extracts, and the flavonoid with the highest content was rutin, a quercetin glycoside (Table 1). The content of rutin was the highest in DMLEE (44.88 ± 0.436 mg/g), followed by DMLDE (15.723 ± 0.005 mg/g), DMSEE (1.085 ± 0.059 mg/g) and DMSDE (0.122 ± 0.095 mg/g). After rutin, taxifolin was the flavonoid compound with the highest content, which was the highest in DMLEE (11.705 ± 0.029 mg/g), followed by DMLDE (4.369 ± 0.014 mg/g), DMSEE (1.593 ± 0.023 mg/g) and DMSDE (1.253 ± 0.218 mg/g).

All 12 Phenolic acids were identified in the extracts, and the major phenolic acids were caffeic acid and chlorogenic acid, a caffeic acid glycoside. However, there was more caffeic acid in the ethanol extracts than the distilled water extracts, and more chlorogenic acid in the distilled water extracts than the ethanol extracts (Table 2). DMLDE (5.165 ± 0.004 mg/g) contained the most chlorogenic acid, followed by DMLEE (2.945 ± 0.119 mg/g), DMSDE (1.532 ± 0.637 mg/g), and DMSEE (0.811 ± 0.024 mg/g). Caffeic acid content was the highest in DMLEE (21.824 ± 1.356 mg/g), followed by DMLDE (13.850 ± 0.024 mg/g), DMSEE (17.446 ± 0.286 mg/g) and DMSDE (11.072 ± 0.178 mg/g).

### 3.4. Effects of DML Extracts on Suppression of Liver Damage

The effect of DML extracts on liver damage due to alcohol consumption was examined by orally administering DML extract for seven days and then excessive alcohol in a binge rat model [36]. After sacrificing the rats, the activity of serum ALT and AST enzymes were compared as indicators of liver damage, as was liver weight. During the experiment, rats in all groups showed weight gain, and recent studies reported that continuous consumption of excessive alcohol causes fat metabolism in the liver and formation of fatty liver tissue, which results in liver hypertrophy [37]. In this study, the alcohol-administered groups also showed decrease in weight compared to the group that was not administered alcohol. Body and liver weight gain for the group given DML extracts did not show significant difference from that of Ethanol groups (Table S3).

**Figure 1:**
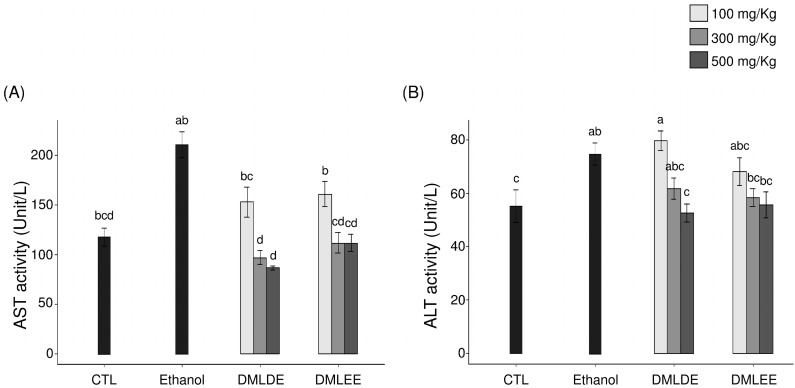
Effect of the *Dendropanax morbifer* leaf ethanol and D.W extracts on serum ALT and AST activities in alcohol-fed rat. (**A**) AST activity and (**B**) ALT activity. The data are expressed as the mean ± SD (*n* = 6), and different letters indicate (a > b > c > d) a significant difference at *p* < 0.05, as determined by a Duncan’s multiple range test.

Serum AST and ALT activities were investigated and used as an index to judge the extent of direct liver damage, as AST and ALT are released from the liver to blood in response to liver damage, increasing their enzymatic activity. Results from Figure 1 show that repeated intake of high concentrations of alcohol was shown to increase serum AST and ALT activities (*p* < 0.05 compared to the Control group). However, the groups with DML extract intakes had inhibited AST and ALT activities after alcohol consumption. In particular, the group that consumed the 500 mg/kg ethanol extract had a 40% decrease in AST activity and a 58% decrease in ALT activity compared to the alcohol intake group. The group that consumed the 500 mg/kg distilled water extract showed AST activity that was reduced by 53% and ALT activity that was reduced by 65% compared to the alcohol intake group.

**Figure 2:**
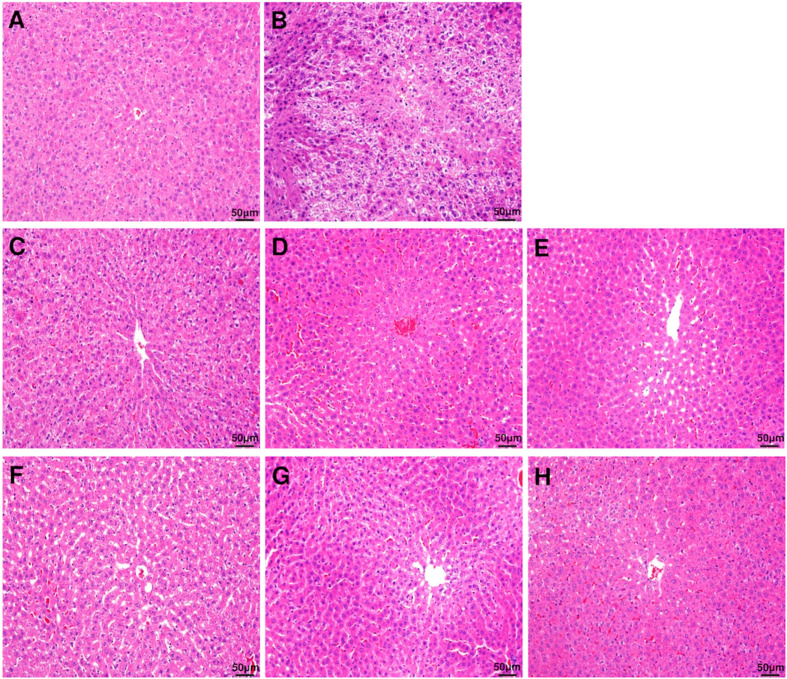
Histopathological evaluation (H&E staining) of rat liver. Liver section images of (**A**) control group, (**B**) ethanol group, (**C**) EtOH + DMLDE 100 mg/kg, (**D**) EtOH + DMLDE 300 mg/kg and (**E**) EtOH + DMLDE 500 mg/kg (**F**) EtOH + DMLEE 100 mg/kg, (**G**) EtOH + DMLEE 300 mg/kg and (**H**) EtOH + DMLEE 500 mg/kg display histopathological changes in liver.

The damage to the liver tissue due to excessive ethanol consumption represented by histochemical staining of the liver is shown in Figure 2. The control group with no ethanol consumption showed a normal hepatocyte structure, whereas the group with ethanol consumption showed reduced boundaries between the hepatocytes, and enlargement of hepatocytes due to fat accumulation and infiltration of neutrophils were observed. However, the symptoms and the histopathological changes were improved in the groups that were administered with DML extract.

### 3.5. Reduction of Ethanol and Acetaldehyde in Blood with DML Extract Intake

**Figure 3:**
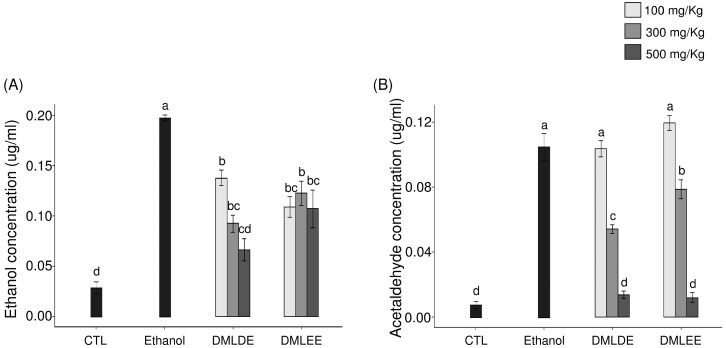
Effect of the *Dendropanax morbifer* leaf ethanol and D.W extracts on serum concentration of ethanol and acetaldehyde. (**A**) Ethanol concentration and (**B**) acetaldehyde concentration. The data are expressed as the mean ± SD (*n* = 6), and different letters indicate (a > b > c > d) a significant difference at *p* < 0.05, as determined by a Duncan’s multiple range test.

Oral administration of excessive alcohol increases absorption of ethanol in the small intestines, and it is metabolized to acetaldehyde through ADH and ALDH in the liver. After liver damage, the decomposition of alcohol by ADH and ALDH decreases, so the concentrations of ethanol and acetaldehyde in blood can be used as an indirect indicator for examining liver damage caused by ethanol. As shown in Figure 3, the group with excessive ethanol intake had 1.8 times higher serum ethanol level (*p* < 0.05) and 2.1 times higher acetaldehyde level than the ethanol group. However, the group being given DML extracts showed a concentration-dependent decrease in blood ethanol and acetaldehyde concentration, and for a given concentration, the administration of the distilled water extracts showed a significant reduction compared to the use of the ethanol extracts.

### 3.6. Effects of DML Extracts on Antioxidant Enzymes in Liver Tissues

**Figure 4:**
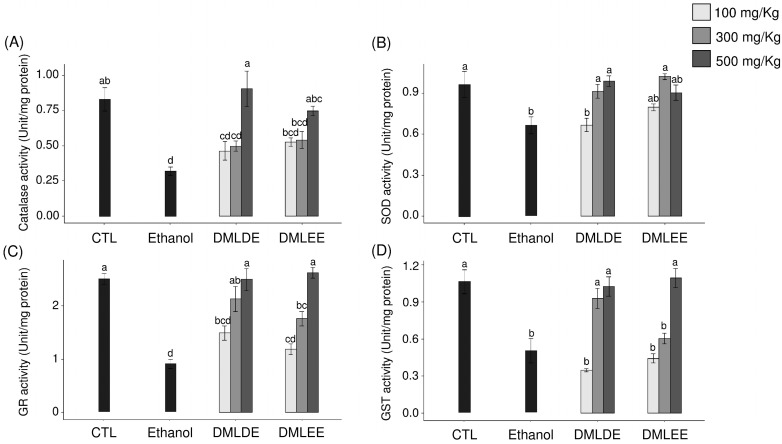
Effects of the *Dendropanax morbifer* leaf D.W and ethanol extracts on antioxidant enzyme activity in alcohol-fed rat. (**A**) Catalase activity, (**B**) SOD activity, (**C**) GR activity and (**D**) GST activity. The data are expressed as the mean ± SD (*n* = 6), and different letters indicate (a > b > c > d) a significant difference at *p* < 0.05, as determined by a Duncan’s multiple range test.

ROS produced by continuous alcohol intake weakens the defense mechanisms associated with oxidative stress in the body and induces oxidative damage. Therefore, the impact of DML extract intake on the activities of antioxidant enzymes related to other antioxidant defenses were evaluated. Figure 4 shows the activities of the antioxidant enzymes, CAT, SOD, GST, and GR. Among the ROS produced in the body, superoxide is converted to hydroxyl radicals by SOD, and the produced hydroxyl radicals are decomposed into water by CAT. CAT activity decreased in the control group compared to the control group, but with no significant difference. In addition, groups with 100 or 300 mg/kg intakes of DML extract did not show differences in CAT activity compared to the ethanol group, but the group with an intake of 500 mg/kg had significantly increased CAT activity. SOD activity was significantly decreased in the ethanol group compared to the control group, and the enzyme activity significantly increased with the intake of DML extracts.

### 3.7. Microbiota Shifted by DML Extracts

**Figure 5:**
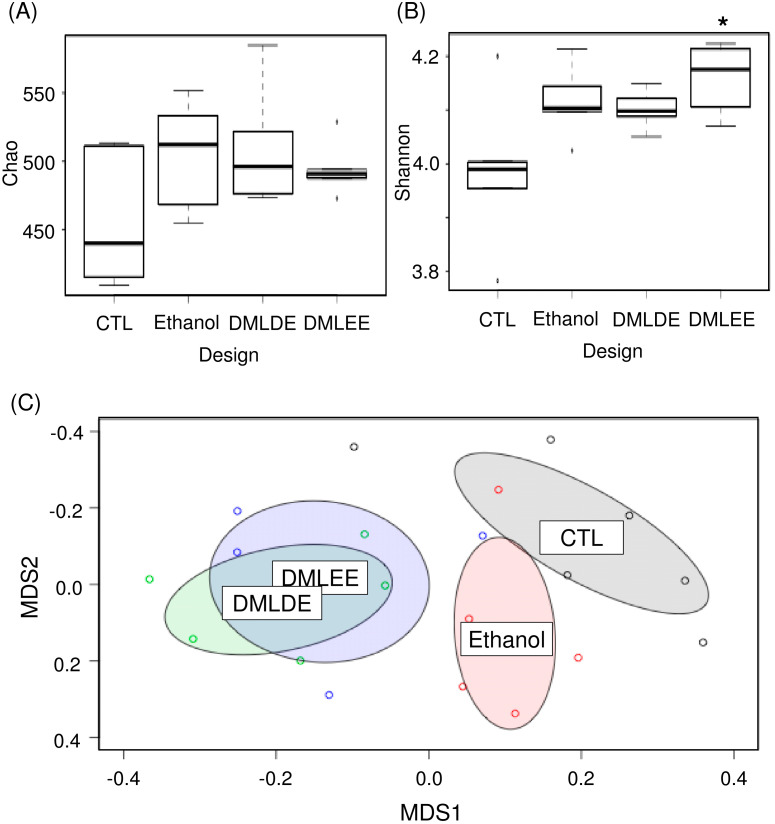
The comparative analysis of gut microbiota using ecological indices and non-metric multidimensional scaling (NMDS). (**A**) Species richness, (**B**) species evenness, and (**C**) NMDS. * indicates significant difference between CTL and DMLEE.

Various concentrations of DML extracts affected microbiota similarly except for DMLEE at 300 mg/kg and DMLDE at 100 mg/kg (Figure S3). With a few exceptions, most of the DML treatments did not show significant differences between the different types of extracts nor concentrations of DML extracts (Table S4). Among the treatments, we selected rats treated with highest concentrations of DMLDE and DMLEE for the further gut microbiota analyses, because they showed most distinctive improvements from the liver damages. Results from Figure 5 show that species evenness between the CTL and DMLEE groups was significantly different, while there was no significant difference in species richness. AMOVA and NMDS indicated that no significant microbial community differences between each other across groups before the treatment (Figure S4), but significant differences has detected after the treatment between CTL and Ethanol as well as Ethanol and DML treated samples (Table S4, Figure 5C). These results suggest that alcohol ingestion and DML treatments can significantly shift the microbiota of rats, while extraction methods did not affect the total microbiota shifts.

We investigated taxonomic compositions of rats’ gut microbiota at the phylum, family and genus levels (Figure S5). Two major phyla, Bacteroidetes and Firmicutes were detected at the phylum level. At the lower taxonomic levels, the family Prevotellaceae and the genus *Prevotella* were the most abundant in the rats’ gut microbiota. We observed the family Sutterellaceae and the genera *Prevotella* and *Romboutsia* were significantly decrease by alcohol consumption. However, taxonomic composition did not cluster samples according to the treatment groups.

**Figure 6:**
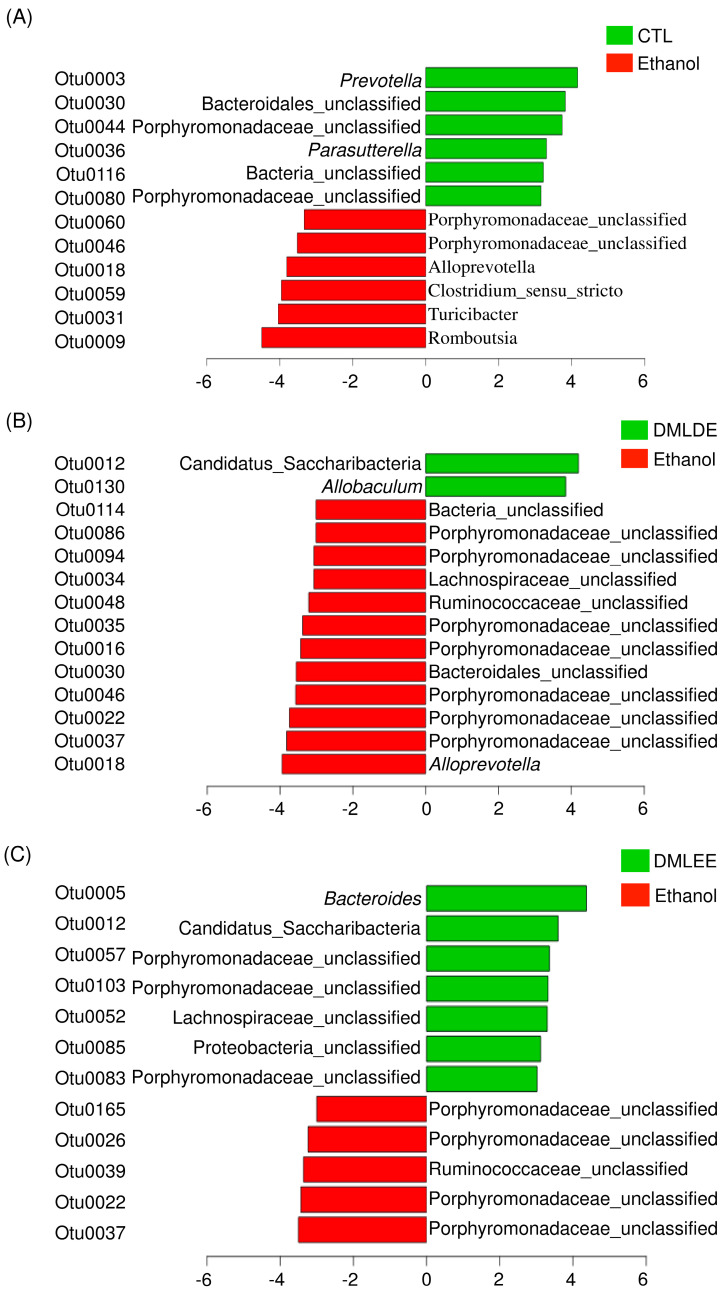
The relative abundance increased or decreased bacteria in OTU level in the comparison between ethanol and CTL or DML extracts. Abundance analysis was performed using linear discriminant analysis effect size (LEfSe). (**A**) Ethanol v/s CTL, (**B**) ethanol v/s DMLDE, (**C**) ethanol v/s DMLEE.

Then differential abundance test was performed to investigate significantly increased or decreased OTUs. Results from Figure 6 show that alcohol consumption significantly increased the abundance of members in the family Porphyromonadaceae, the genus *Alloprevotella, Clostridium, Turicibacter* and *Romboutsia*, while decreased the abundance of other OTUs belonging to the family Porphyromonadaceae, the genus *Prevotella*, and *Parasutterella*. DMLDE increased *Allobaculum* and one unclassified OTU belonging to the phylum Candidatus Saccharibacteria, while decreased 12 OTUs including various unclassified OTUs mostly belonging to the family Porphyromonadaceae. On the other hand, there are seven and five OTUs increased and decreased by DMLEE treatments, respectively. Most of the OTUs were the unclassified members of the family Porphyromonadaceae. Two OTUs (Otu0022 and Otu0037) were decreased and one OTU (Otu0012) was increased by both DML treatments, but other OTUs were specific to each treatment. There were no OTUs whose abundance were changed due to alcohol consumption but recovered by DML treatments.

**Figure 7:**
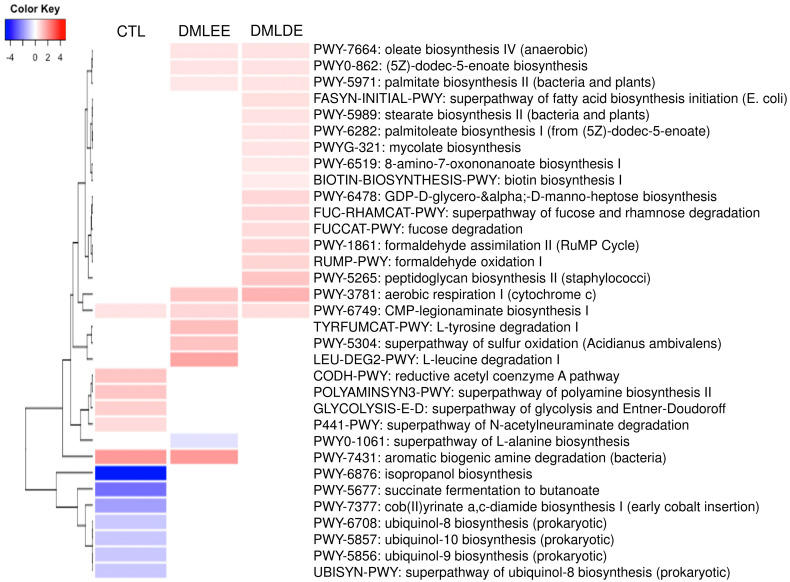
Heatmap analysis of predicted metabolic pathways that were significantly increased or decreased in the comparison between ethanol and CTL or DML extracts. Abundance analysis was performed using ALDEx2 (*p* < 0.05).

PICRUSt2 predicted several metabolic activity changes by alcohol consumption and DML extracts treatments. Results in Figure 7 show that seven and six metabolic activities were predicted to be enriched and depleted due to alcohol consumption, respectively. On the other hand, there are five metabolic activities (biosynthesis of oleic acid, (5Z)-dodec-5-enoate, palmitate, cytochrome c aerobic respiration, and CMP-legionaminate) predicted to be enriched by DML treatments. Beside these, there are four and 12 metabolic activities predicted to be enriched by DMLEE and DMLDE, respectively. Our results showed there were more metabolic activities predicted to be enriched by DMLDE than by DMLEE.

**Figure 8:**
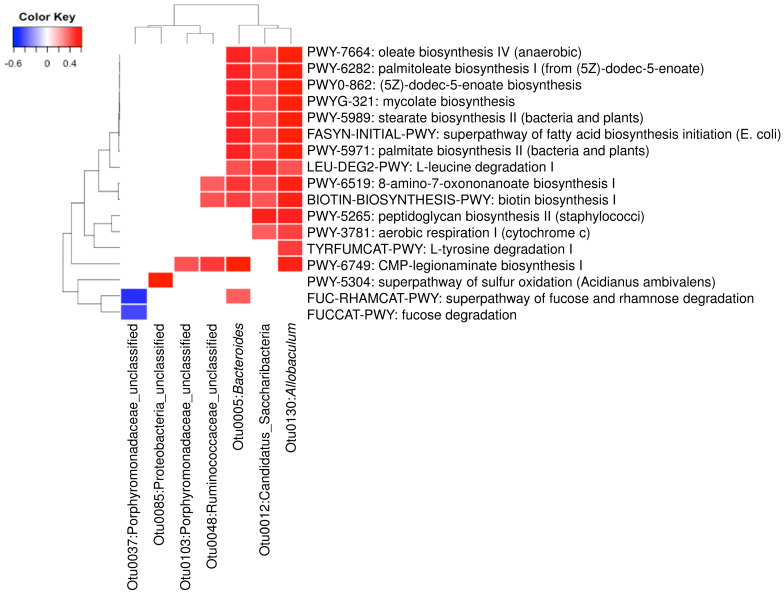
Heatmap analysis of operational taxonomic units (OTUs) associated with the predicted metabolic pathways shifted by DML extracts (*p* < 0.01).

A total of 23 OTUs that were affected by either of both DML treatments in this study. We investigated associations of these OTUs with 21 predicted metabolic activities that were also affected by DML treatments. Results from Figure 8 show that seven OTUs were significantly correlated with one or more of the 17 predicted metabolic pathways. Three OTUs (Otu0005, Otu0012, and Otu0130) were associated with the most of the predicted metabolic pathways, while the other OTUs were associated with only one or two. The three OTUs are classified to be the genera *Bacteroides* and *Allobaculum* and one unclassified belonging to the phylum Candidatus Saccharibacteria. Predicted metabolic pathways associated with these OTUs were mostly related with monounsaturated fatty acids (oleate and palmitoleate) synthesis pathways.

## 4. Discussion

The liver is an organ that plays an important role in the metabolism of various toxic substances, including ethanol. In the body, 80–90% of alcohol is first decomposed into acetaldehyde by ADH present in liver cells, then metabolized by ALDH to form acetic acid, which finally undergoes complete decomposition via hydrolysis into carbon dioxide and water. Nicotinamide adenine dinucleotide phosphate (NADP), acetaldehyde, and various ROS produced during alcohol metabolism in the liver react with DNA and proteins to act as major mediators of liver damage [38]. Therefore, various antioxidants such as plant extracts have been recently suggested for use as medicines and functional foods to prevent liver damage caused by oxidative stress by inhibiting ROS production due to ethanol [39]. It was also found in several previous studies that DML and branch extracts have strong antioxidant activities, and so their function for reducing liver damage was studied. First, it was confirmed that the antioxidant activity of DML extract was higher than that of the stem extract. Antioxidant activity showed a close relationship to the total phenol and flavonoid content in the extract [17,19]. Total phenolic compounds have a hydroxyl group attached to an aromatic ring, while flavonoids have a basic C6-C3-C6 skeleton [40]. These compounds have a common aromatic hydroxyl group with excellent ability to donate electrons, with donation being stabilized by a resonance effect, leading to excellent antioxidant activity [41]. The antioxidant activity of DML extracts in this study may also be due to the effects of these total phenolics and flavonoids. Previous studies on the effect of harvesting time on the antioxidant activities of DML extracts showed that antioxidant activity was correlated with total phenolic and flavonoid content, and that DML extract had greater antioxidant activity than the branch or bark extracts, which was consistent with the findings of this study [17]. We measured the prevention of liver damage due to ethanol using DML extracts with excellent antioxidant activity, and found that both ethanol and distilled water extracts prevented liver damage, with the latter showing a much greater effect as opposed to in vitro experiments.

Chronic ethanol intake promotes liver damage by reducing the activity of antioxidants such as glutathione (GSH) and various antioxidant enzymes in the body [5]. The effects of GST and GR related to the metabolism of GSH on the enzymatic activity were confirmed. GSH, which acts as an antioxidant in the body, reacts with radicals via GST to form inactive glutathione disulfide (GSSG), with the cycle then closing via conversion of GSSG back into active GSH by GR [42]. Both GST and GR activities were significantly decreased in the Ethanol group compared to the control group, but the intake of DML extract led to the recovery of their activities up to that of the control group. In addition, DMLDE showed a greater effect than DMLEE groups of the same doses.

Activities of antioxidant enzyme such as SOD, GST, and GR were reduced in the Ethanol group compared to the control group, and the groups that were administered DML extracts showed a concentration-dependent increase in the activities of all antioxidant enzymes. In a study by Bae et al. [21], subjects were given 100 or 300 mg/kg of DMLDE for four weeks and ethanol for the next three weeks, and it was found that the activities of antioxidant enzymes related to glutathione metabolism were reduced in the EtOH-treated group. For the treatment group that received 300 mg/kg of DMLDE, a similar recovery of antioxidant activity was observed to that seen for a positive control group treated with silymarin.

Plant-derived flavonoids and phenolic acids have strong antioxidant properties and are known to prevent inflammatory diseases, cancer, cardiovascular diseases, and various degenerative diseases associated with oxidative stress [13,43]. Previous studies have found that rutin and chlorogenic acid are the main components of *D. morbifera* leaves, which is consistent with the findings of this study [19]. Rutin in particular has been reported to exhibit anti-inflammatory activity against various inflammatory diseases and to protect the liver from various liver damage models [44]. Intake of chlorogenic acid, a type of caffeic acid glycoside that is present in coffee beans, is known to prevent liver damage caused by ethanol consumption by increasing inflammation and antioxidant enzymatic activities in liver [45]. In this study, the rutin, chlorogenic acid, and caffeic acid that are present in the extract are known to prevent liver damage.

In this study, we observed that alcohol consumption increased *Prevotella* and *Parasutterella* and decreased *Clostridium*, *Turcibacter* and *Romboutsia*, which is in line with the previous reports [46,47,48,49]. Moreover, alcohol consumption also increased the abundance of some of the gram-negative bacteria (i.e, Porphyromonadaceae and *Alloprevotella*). It has been reported that alcohol consumption may compromise intestinal permeability, allowing the intestinal lipopolysaccharide (LPS) to flow into the liver through the portal vein [50]. In contrast, treatment of DML extracts decreased relative abundance of these gram-negative bacteria. Moreover, it has been reported that DML extracts suppressed the production of LPS-induced pro-inflammatory mediators and cytokines [51] We observed that DMLDE increased the genus *Allobaculum*, known as a butyrate producer [52]. Butyrate was reported to improve alcohol-derived liver damage through enhancing intestinal epithelial barrier function in mice [53,54]. On the other hand, DMLEE increased the abundance of OTUs belonging to the family Porphyromonadaceae and *Bacteroides* and decreased the abundance of other members of the family Porphyromonadaceae. Previous study has reported that gut microbiota of mice ingested with pectin restored *Bacteroides* and completely prevented alcohol-derived liver damage, suggesting that abundance of *Bacteroides* may be associated with the recovery from alcohol-derived liver damage [55]. Although many members of the family Porphyromonadaceae include short chain fatty acids producers such as *Butyricimonas*, *Coprobacter* and *Macellibacteroides* [56,57,58], this family has been reported to be associated with alcohol consumption and resulting complications in chronic liver diseases [59,60]. To date, due to the lack of genetic information, further studies are required at the genus or species level to investigate roles of these OTUs belonging to the family Porphyromonadaceae.

Our data suggested that isopropanol biosynthesis and succinate fermentation to butanoate may have been largely enriched by alcohol consumption due to secondary alcohol dehydrogenase and succinate semialdehyde produced by *clostridium* strains. Ubiquitous bacteria produce ubiquinone which can be used to catalyze ethanol to a ubiquinol and acetaldehyde. Therefore, there is a possibility that the alcohol consumption may have enriched the metabolic activities of bacterial ubiquinol biosynthesis. Cmp-legionaminate biosynthesis was also depleted by alcohol consumption, but our data also suggested DML treatments may have recovered both of them. The legionaminic acid has been reported to be a virulence-associated cell-surface glycoconjugate in *Campylobacter*, an intestinal pathogenic bacteria [61], however another study has reported that this pathway was also predicted to be enriched in the cecum of probiotics treated broilers [62]. In our data, DMLEE showed enriched aromatic biogenic amine degradation which was depleted by alcohol consumption. Many of aromatic biogenic amines are involved in gastrointestinal pathology such as intestinal bowel syndrome and inflammatory bowel disease [63]. In this study, our results suggested that this biogenic amine degradation could be associated with the enrichment of L-tyrosine degradation. Besides Cmp-legionaminate biosynthesis, there were four more enriched metabolic pathways by both DML treatments, including aerobic respiration I (cytochrome c), oleate biosynthesis IV, (5z)-dodec-5-enoate biosynthesis, and palmitate biosynthesis II. Alcohol consumption is known to be negatively associated with activity of mitochondria [64], thus enriched metabolic activities of cytochrome c suggest that DML treatments may have reduced the damage caused by alcohol consumption. The rest of the three enriched metabolic activities by DML treatments were similar to each other, suggesting these might be predicted from the abundance of similar bacterial species. Oleate and palmitoleate are the major monounsaturated fatty acids in olive oils and its consumption has been reported to improve liver secretory activity, reduce inflammation and oxidative stress [65]. Our data indicated that DMLDE may have enhanced the metabolic activities of biosynthesis of stearate, palmitoleate, and biotin. Stearate is converted to oleate by stearoyl-CoA desaturase 1 (SCD1) and palmitoleate is produced from palmitate, therefore enhancement of these metabolic pathways may bring similar benefits as olive oils do. It has been reported that the amount of SCD1 is substantially low in germ free mice, suggesting that SCD1 is regulated by gut microbiota [66]. Biotin biosynthesis was also predicted to be enhanced by DMLDE. Biotin is a B-complex vitamin that acts as an essential coenzyme. Low biotin levels can occur in elderly individuals, excessive alcohol consumers and smokers. Glucokinase decreased by alcohol consumption can be increased by supplementation of biotin [67,68]. DMLDE also enriched fucose degradation, suggesting increased intestinal bacteria that consume fucose. Fucose is a monosaccharide abundant in mammalian gut, and a degradation of fucose plays an important role in maintaining gut homeostasis resulting in reduced inflammation and controlled hepatic bile acid synthesis [69]. Collectively, our data suggest that DML extracts, especially DMLDE may have enriched metabolic activities predicted to be beneficial to the gut and liver.

Furthermore, we investigated the association of the differentially abundant bacteria with the predicted metabolic activities. We observed that many of the predicted metabolic changes were positively associated with *Bacteroides*, *Allobaculum* and one OTU of Candidatus Saccharibacteria. Previously, it has been reported alcoholic patients have significantly low abundance of *Bacteroides* in human gut [70]. Xiao et al. [71] reported that chronic alcohol consumption shifts gut microbiota which may be a partial cause of alcohol withdrawal-induced anxiety. The decreased abundance of *Allobaculum* was the most obvious shift caused by excessive alcohol consumption. Our results suggest that DML extracts significantly increased the abundance of these genera, suggesting that DML extracts may partially recover the gut microbiota shifted by alcohol consumption. Millman et al. [72] reported that the ingestion of extra virgin olive oils significantly increased these two genera. In addition, they reported that the abundance of *Bacteroides* was found to be associated with loss of plasma triglyceride concentration. Houghton et al. [73] suggested that *Allobaculum* may improve age-related mitochondrial dysfunction through producing butyrate. With the increasing evidence of beneficial effects of these bacteria, our results suggest that DML extracts may improve intestinal functions through shifting gut microbiota impaired by alcohol consumption.

In this study, we investigated antioxidative effects of DML extracts on rats fed ethanol. Our results showed that DML extracts significantly improve the damage cause d by alcohol consumption. However, there are limitations in the study. Metabolomic shifts were only estimated by gut microbiota shifts using PICRUSt2, which could be more accurate if mass-spectrometry metabolomics was applied. In addition, further study should proceed for the identification of bioactive compounds in the DML extracts. Nevertheless, the study presents the effects of DML extracts not only on rats’ physiology but also on gut microbiota, providing a fundamental information of how DML extracts may act as functional food.

## 5. Conclusions

Summarizing these results, we believe that the large amounts of polyphenol compounds contained in DML extracts promote ethanol metabolism in the liver, preventing liver damage. Our results showed hot water extracts showed better improvement in vivo, suggesting that water extracts may provide more bioavailable materials, resulting in enriching beneficial intestinal metabolic activities. These results suggest that DML extracts, especially ones extracted with hot water, may be used as a material for new functional foods or medicines that can prevent and treat liver toxicity caused by ethanol.

## Figures and Tables

**Table 1:** Individual flavonoid contents in the *Dendropanax morbifer* leaf and stem extracts (unit: ug/g of extracts).

Flavonoids	DMLDE	DMLEE	DMSDE	DMSEE
Flavonoid glycoside (mg/g of extracts)				
Naringin	0.848 ± 1.075	0.017 ± 0.014	0.116 ± 0.010	0.104 ± 0.184
Narirutin	0.176 ± 0.012	2.808 ± 0.015	0.329 ± 0.117	0.227 ± 0.173
Neohesperidin	0.185 ± 0.003	3.308 ± 0.009	0.351 ± 0.149	0.053 ± 0.037
Hesperidin	0.021 ± 0.002	1.256 ± 0.179	0.484 ± 0.003	0.374 ± 0.004
Rutin	15.723 ± 0.005	44.88 ± 0.436	0.122 ± 0.095	1.085 ± 0.059
Flavonoid aglycone (mg/g of extracts)				
Apigenin	-	-	-	-
Hesperetin	-	-	-	-
Isorhaemnetin	0.428 ± 0.001	-	0.385 ± 0.076	0.282 ± 0.096
Kaempferol	-	-	-	-
Luteorin	-	-	-	-
Myricetin	0.245 ± 0.001	0.306 ± 0.001	0.236 ± 0.004	0.203 ± 0.001
Naringenin	0.546 ± 0.007	-	0.406 ± 0.013	0.499 ± 0.034
Quercetin	0.149 ± 0.002	0.148 ± 0.001	0.020 ± 0.001	0.139 ± 0.001
Rhaemnetin	-	-	-	-
Taxifolin	4.369 ± 0.014	11.705 ± 0.029	1.253 ± 0.218	1.593 ± 0.023
Polymethoxyflavone (mg/g of extracts)				
Nobiletin	0.615 ± 0.012	0.574 ± 0.001	-	-
Sinesetin	0.542 ± 0.001	0.461 ± 0.073	-	-
Tangeretin	0.265 ± 0.002	0.579 ± 0.095	-	-

Means ± SD of determinations were made in triplicate experiments.

**Table 2:** Individual phenolic acid contents in the *Dendropanax morbifer* leaf and stem extracts (unit: ug/g of extracts).

Derivatives	DMLDE	DMLEE	DMSDE	DMSEE
Benzoic acid derivative (mg/g of extracts)				
Benzoic acid	0.460 ± 0.002	1.081 ± 0.022	0.702 ± 0.073	0.638 ± 0.033
*p*-Hydroxybenzoic acid	0.294 ± 0.146	0.196 ± 0.040	0.167 ± 0.016	0.153 ± 0.003
Protocatechuic acid	0.663 ± 0.001	0.553 ± 0.001	0.466 ± 0.187	0.248 ± 0.001
Vanillic acid	0.611 ± 0.322	0.333 ± 0.053	0.843 ± 0.086	0.966 ± 0.045
Syringic acid	0.297 ± 0.002	0.371 ± 0.115	1.054 ± 0.534	0.454 ± 0.045
Gallic acid	0.653 ± 0.007	0.732 ± 0.003	0.597 ± 0.062	0.364 ± 0.315
Cinnamic acid derivative (mg/g of extracts)				
Cinnamic acid	0.164 ± 0.001	0.105 ± 0.001	0.161 ± 0.043	0.111 ± 0.001
*p*-Coumaric acid	0.679 ± 0.018	2.092 ± 0.247	0.654 ± 0.178	0.892 ± 0.078
Caffeic acid	13.850 ± 0.024	21.824 ± 1.356	11.072 ± 0.178	17.446 ± 0.286
Ferullic acid	0.440 ± 0.001	0.916 ± 0.003	0.452 ± 0.020	0.475 ± 0.006
Sinapinic acid	0.417 ± 0.001	0.359 ± 0.001	0.428 ± 0.016	0.563 ± 0.001
Chlorogenic acid	5.165 ± 0.004	2.945 ± 0.119	1.532 ± 0.637	0.811 ± 0.024

Means ± SD of determinations were made in triplicate experiments.

## Supplementary Materials

The following are available online at https://www.mdpi.com/2076-3921/9/10/911/s1, Figure S1: Animal experiment design, Table S1: Total polyphenol and total flavonoid contents of *Dendropanax morbifer* leaf and stem extracts, Table S2: Antioxidant activity of the *Dendropanax morbifer* leaf and stem extracts, Figure S2: HPLC chromatogram of *Dendropanax morbifer* leaf and stem extracts, Table S3: Body weight change and liver-body weight ratio of *Dendropanax morbifer* leaf ethanol and D.W extracts in alcohol-fed rats, Figure S3: Comparison of microbiota according to the concentrations of DML extracts. Numbers indicate the concentrations of DML treatments, Figure S4: Comparison of microbiota before DML extract treatment, Table S4: Analysis of molecular variances (AMOVA), Figure S5: Comparison of the taxonomic composition.
