# Supplementary material for: Dendropanax morbifera Leaf Extracts Improved Alcohol Liver Injury in Association with Changes in the Gut Microbiota of Rats
Source: Antioxidants (Basel). 2020 Sep 24;9(10):911. doi: 10.3390/antiox9100911 (PMC7598590; doi:10.3390/antiox9100911)
Supplement: Supplementary file 1 [file antioxidants-09-00911-s001.pdf]

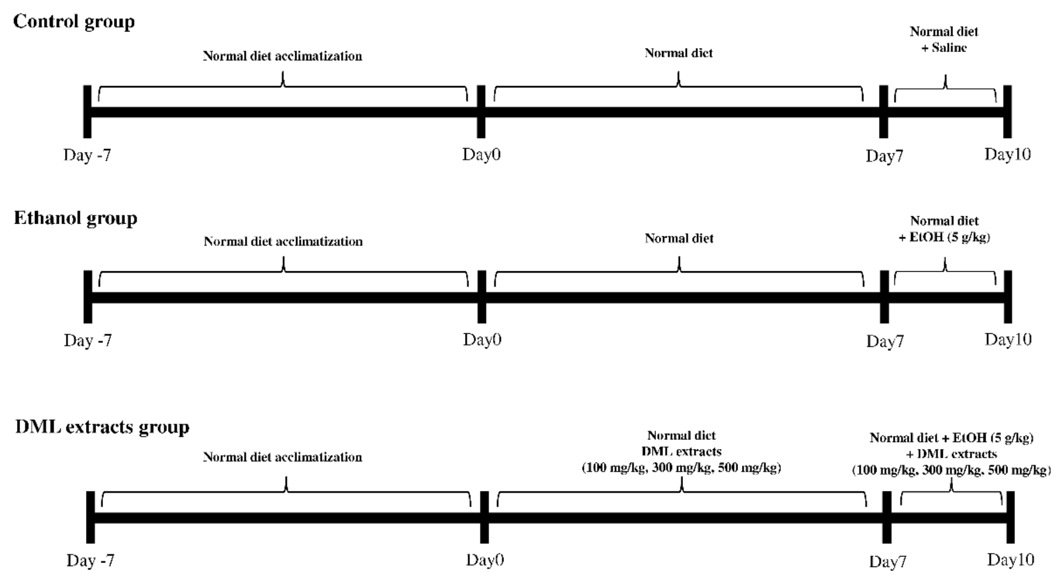

Figure S1. Animal experiment design.

Table S1. Total polyphenol and total flavonoid contents of *Dendropanax morbifer* leaf and stem extracts.

	D. morbifera **leaf**		***D. morbifera*** **sterm**	
	D.W	70% EtOH	D.W	70% EtOH
Total poly phenol (mg/g extracts)	52.41±0.41	54.17±0.34	32.04±0.76	35.10±0.41
Total flavonoid (mg/g extracts)	14.07±0.12	19.47±0.14	1.95±0.04	4.90±0.08

Means ± SD of determinations were made in triplicate experiments.

Table S2. Antioxidant activity of the *Dendropanax morbifer* leaf and stem extracts.

	D. morbifera **leaf**		***D. morbifera*** **sterm**	
	D.W	70% EtOH	D.W	70% EtOH
DPPH radical scavenging (IC_{50})	16.52±0.30	15.61±0.19	46.46±1.34	36.02±1.78
FRAP (mM $FeSO_4$/g)	617.51±5.25	623.10±2.77	303.02±3.99	297.12±0.88
TEAC (mM Trolox/g)	188.24±0.86	187.21±3.99	136.94±1.85	125.71±1.74

Means ± SD of determinations were made in triplicate experiments.

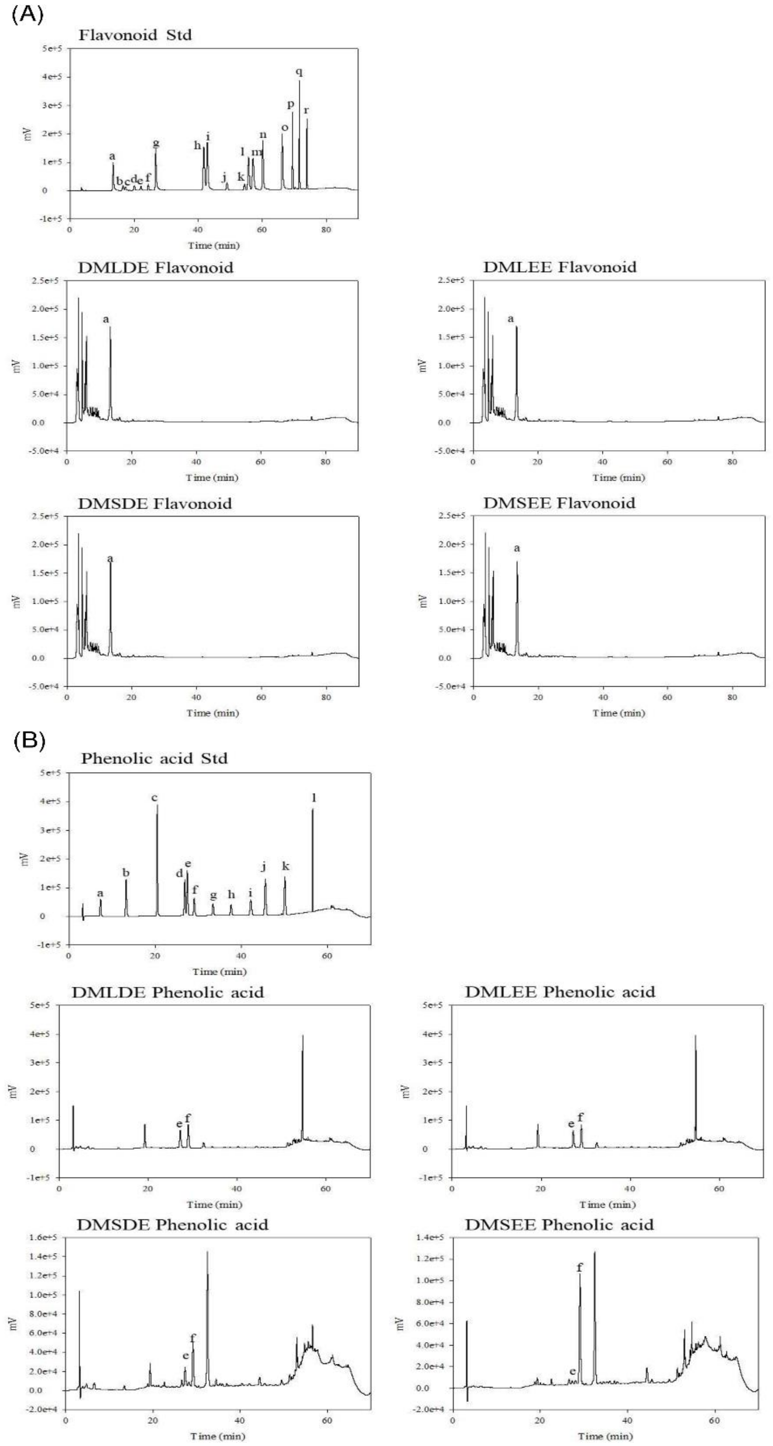

Figure S2. HPLC chromatogram of *Dendropanax morbifer* leaf and stem extracts. (A): Flavonoid HPLC chromatographic profiles of *Dendropanx morbifera* leaf and stem D.W and ethanol extracts. Compounds identified in the figures are (a) rutin, (b) taxifolin, (c) narirutin, (d) naringin, (e) hesperidin, (f) neohesperodin, (g) myricetin, (h) quercetin, (i) luteolin, (j) naringenin, (k) hesperetin, (l) Apigenin, (m) kaempferol, (n)isorhaemnetin, (o) rhaemnetin, (p) sinensetin, (q) nobiletin and (r) tangeretin. (B): Phenolic acid HPLC chromatographic profiles of Dendropanx morbifera leaf and stem D.W and ethanol extracts. Compounds identified in the figures are (a) gallic acid, (b) protocatechuic acid, (c) p-hydroxybenzoic acid, (d) vanillic acid, (e) chlorogenic acid, (f) caffeic acid, (g) syringic acid, (h) p-coumaric, (i) benzoic acid, (j) ferullic acid, (k) sinapinic, and (l) cinnamic.

Table S3. Body weight change and liver-body weight ratio of *Dendropanax morbifer* leaf ethanol and D.W extracts in alcohol-fed rats.

	CTL	Ethanol	DMLDE			DMLEE		
			100	300	500	100	300	500
Initial Body weight (g)	238.69±30.19	237.99±29.84	234.83±34.10	233.86±37.88	232.27±39.55	236.64±28.30	237.44±27.54	236.85±28.68
Final body weight (g)	292.68±29.92	275.27±25.04	269.90±31.53	269.51±44.87	280.40±27.26	262.29±31.42	287.66±24.52	292.27±21.27
Liver weight (g)	10.83±2.00	10.47±0.98	9.88±1.10	9.90±1.43	9.76±0.58	9.71±1.06	11.20±1.06	10.88±1.13
Body weight liver ratios (%)	3.69±0.53	3.20±1.29	3.27±1.25	3.27±1.26	3.23±1.25	3.88±0.34	3.81±0.22	3.25±1.24

Means ± SD of determinations were made in triplicate experiment

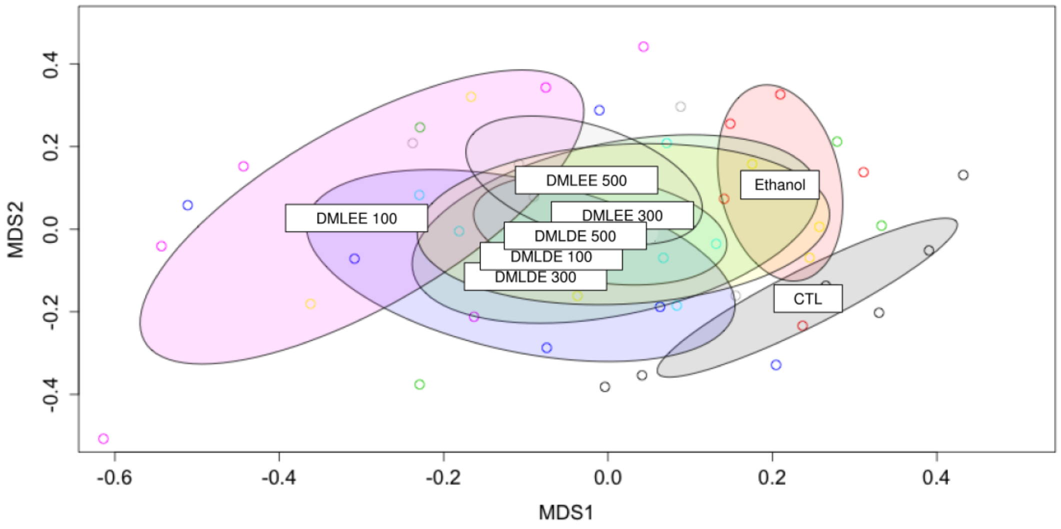

Figure S3. Comparison of microbiota according to the concentrations of DML extracts. Numbers indicate the concentrations of DML treatments.

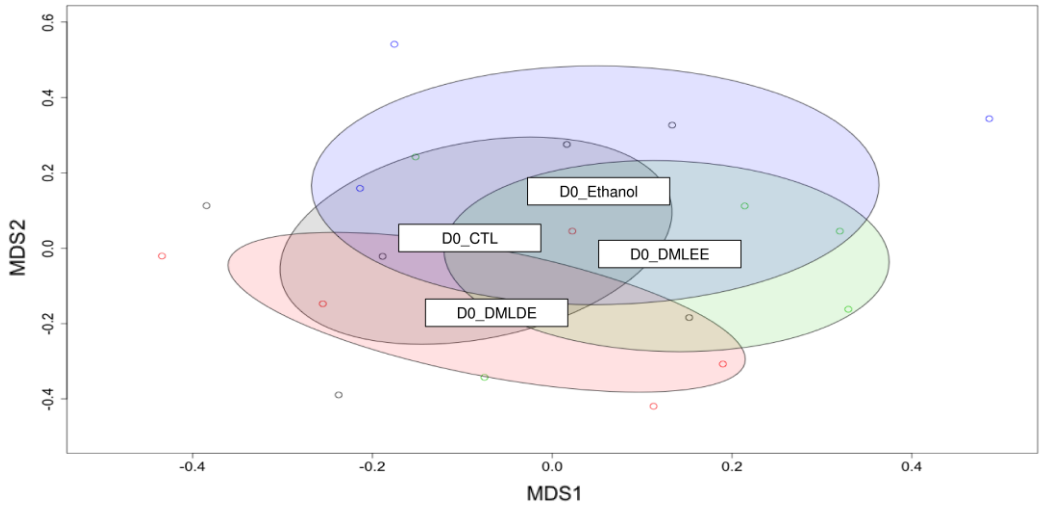

Figure S4. Comparison of microbiota before DML extract treatment.

Table S4. Analysis of molecular variances (AMOVA)

Group (Fs)	CTL	Ethanol	DMLDE 100	DMLDE 300	DMLDE 500	DMLEE 100	DMLEE 300	DMLEE 500
CTL	-	1.660*	1.403	1.794*	2.176**	2.899**	1.402	1.872**
Ethanol	1.660*	-	1.362	1.920**	2.174**	2.409*	1.403	1.695*
DMLDE 100	1.403	1.362	-	1.102	1.220	1.901	0.847	0.751
DMLDE 300	1.794*	1.920**	1.102	-	1.202	1.497	1.105	1.298
DMLDE 500	2.176**	2.174**	1.220	1.202	-	2.409*	1.233	1.125
DMLEE 100	2.899**	2.409*	1.901	1.497	2.409*	-	1.718	2.131*
DMLEE 300	1.402	1.403	0.847	1.105	1.233	1.718	-	0.846
DMLEE 500	1.872**	1.695*	0.751	1.298	1.125	2.131*	0.846	-

* $P < 0.05$ and ** $p < 0.001$

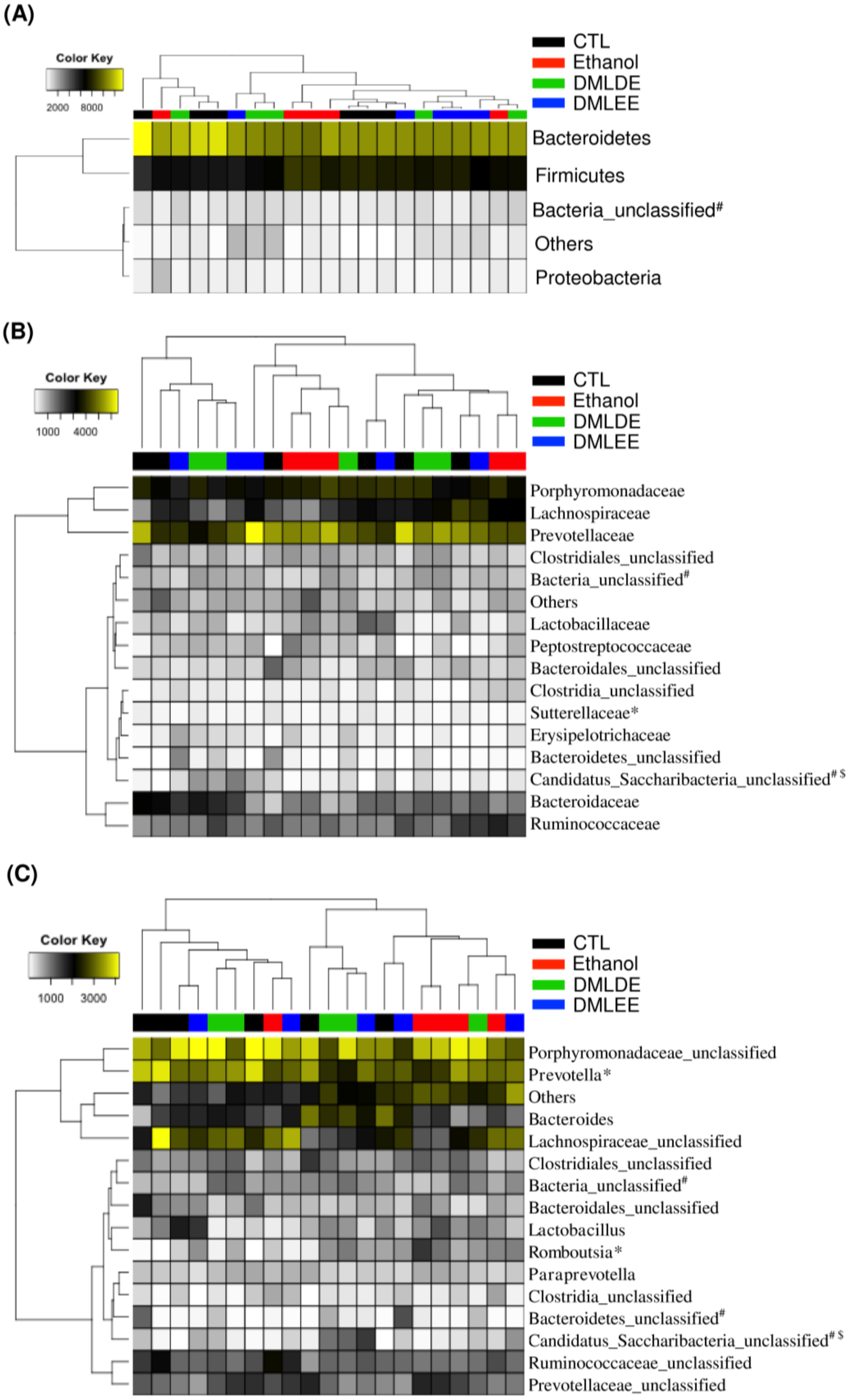

Figure S5. Comparison of the taxonomic composition. (A) Phylum, (B) Family and (C) Genus level. *, # and $ indicate significant difference between Ethanol and CTL, Ethanol and DMLDE, and Ethanol and DMLEE, respectively ($p < 0.05$). The relative abundance was examined using LEfSe analysis.
